# Autosomal monoallelic expression in the mouse

**DOI:** 10.1186/gb-2012-13-2-r10

**Published:** 2012-02-20

**Authors:** Lillian M Zwemer, Alexander Zak, Benjamin R Thompson, Andrew Kirby, Mark J Daly, Andrew Chess, Alexander A Gimelbrant

**Affiliations:** 1 Center for Human Genetic Research, Massachusetts General Hospital, Boston, MA 02114, USA; 2 Department of Cancer Biology, Dana-Farber Cancer Institute, 450 Brookline Ave, Boston, MA 02115, USA; 3 Department of Developmental and Regenerative Biology, Fishberg Department of Neuroscience, Department of Genetics and Genomic Sciences, Friedman Brain Institute, Mount Sinai School of Medicine, New York, NY 10029, USA; 4 Department of Genetics, Harvard Medical School, Boston, MA 02115, USA; 5 Current address: Pfizer Research Business Technologies, 35 Cambridgepark Drive, Cambridge, MA 02140, USA

## Abstract

**Background:**

Random monoallelic expression defines an unusual class of genes displaying random choice for expression between the maternal and paternal alleles. Once established, the allele-specific expression pattern is stably maintained and mitotically inherited. Examples of random monoallelic genes include those found on the X-chromosome and a subset of autosomal genes, which have been most extensively studied in humans. Here, we report a genome-wide analysis of random monoallelic expression in the mouse. We used high density mouse genome polymorphism mapping arrays to assess allele-specific expression in clonal cell lines derived from heterozygous mouse strains.

**Results:**

Over 1,300 autosomal genes were assessed for allele-specific expression, and greater than 10% of them showed random monoallelic expression. When comparing mouse and human, the number of autosomal orthologs demonstrating random monoallelic expression in both organisms was greater than would be expected by chance. Random monoallelic expression on the mouse autosomes is broadly similar to that in human cells: it is widespread throughout the genome, lacks chromosome-wide coordination, and varies between cell types. However, for some mouse genes, there appears to be skewing, in some ways resembling skewed X-inactivation, wherein one allele is more frequently active.

**Conclusions:**

These data suggest that autosomal random monoallelic expression was present at least as far back as the last common ancestor of rodents and primates. Random monoallelic expression can lead to phenotypic variation beyond the phenotypic variation dictated by genotypic variation. Thus, it is important to take into account random monoallelic expression when examining genotype-phenotype correlation.

## Background

In diploid eukaryotic organisms, the maternally and paternally derived copies of each gene are usually assumed to be simultaneously expressed at similar levels. In some cases, however, only one allele is transcribed, while the other allele is transcriptionally silent. These monoallelically expressed genes belong to three separate classes. In the first class, parent-of-origin imprinting (as is the case for *Igf2 *and *H19*), monoallelic expression is determined by marks placed during gametogenesis, which lead to imprinting either in specific tissues or throughout the entire organism [1]. All cells in which a given gene is imprinted have the same active allele, which is determined solely by the parent of origin of the allele. The remaining two classes of genes both fall into the category of random monoallelic expression (RMAE) and include X-chromosome inactivated genes, for which there is chromosome-wide coordination, and autosomal RMAE. In both cases of RMAE genes, the initial random choice between alleles is followed by a stable mitotic transmission of monoallelic expression. In the case of X-inactivation, a random choice is made in individual cells early in female development. This choice affects nearly all genes on one X-chromosome, resulting in inactivation of one copy of the X-chromosome in each cell, and thus monoallelic expression of X-linked genes in every cell of the organism [2].

For a number of individual autosomal genes, a similar random choice, with subsequent maintenance, has been described. This class of autosomal monoallelic expression genes was long thought to consist of isolated examples specific to the immune or nervous systems, including odorant receptor genes, and genes encoding the immunoglobulins, T-cell receptors, interleukins, and natural killer cell receptors [3-9]. However, we have recently shown that a surprisingly large number of human genes with diverse functions (nearly 10% of approximately 4,000 assessed genes) are subject to this type of random monoallelic expression [10].

For autosomal RMAE, as with X-chromosome inactivation, each cell within a given tissue reflects a choice to activate one or both alleles. Unlike the chromosome-wide inactivation observed in X-inactivation, for autosomal genes the allelic choice for each gene is made independently. Individual genes may be monoallelic in some cells and biallelic in others. Distinct clonal cell lineages can each have an apparently unique combination of active and inactive alleles, contributing to a hitherto unsuspected level of epigenetic heterogeneity among genetically and developmentally matched cells [10]. Notably, the designation of this monoallelic expression as random does not indicate a rapid switching of allelic expression within cells. Rather, during development the choice of allelic activity is set and then stably inherited by all daughter cells [9]. While for imprinted genes allele-specific expression can be observed in tissue samples, for X-chromosome and autosomal RMAE, the allele-specific behavior is most readily observed in either single cells or clonal cell lines.

The widespread autosomal RMAE found in humans leads to the question of the extent of RMAE in other mammals, and thus its evolutionary conservation. Scattered examples of autosomal RMAE have been observed and validated in various mammals besides humans. Examples include the allelic exclusion of immunoglobulins in rabbits, rats, and mice [5,6], the monoallelic expression of olfactory receptors in freshly isolated neurons in mice [3], and five additional genes interspersed within the olfactory receptor gene clusters that were found to show RMAE in mouse clonal neural stem cell lines [11]. Additional examples included *Il4*, which has been observed as monoallelic in mouse in both fresh cells and cell culture [7,12,13]. However, a genome-scale analysis of random monoallelic expression in a species other than humans has not been reported. We therefore explored whether the extent and identity of RMAE genes seen in mouse is similar to that documented for humans.

## Results

As the choice of allelic expression is made on a cell-by-cell basis, autosomal RMAE is, for the most part, not observable in non-clonal populations of cells or in whole tissues. This is similar to what is widely known for X-inactivation. With this in mind, we isolated single Abelson murine leukemia virus (A-MuLV) transformed pre-B lymphoblastoid cells using fluorescence-activated cell sorting (Materials and methods; Table S1 in Additional file 1), and cultured them to create clonal cell lines. Use of this cell type allows for comparison with the analyses of human RMAE, which primarily involved B-lymphoblastoid cells [10]. We also used SV-40 large T antigen transformed fibroblasts to establish clonal and nonclonal fibroblast lines (see Materials and methods). For the remainder of this paper, unless otherwise noted, the results described are based exclusively on the lymphoblast clones, which were more thoroughly assessed. Complete results for the fibroblast clones can be found in Additional file 1 (Figure S1, Note 1) and Additional file 2 (Table S2).

We used cells from two distinct mouse crosses to establish the lymphoblast lines. One cross, 129S1/SvImJ dam × Cast/EiJ sire F1, has an intermediate density of heterozygous SNPs with even distribution over the whole genome. We also used reciprocal crosses of Balb/cByJ × C57BL/6J, which have a high density of heterozygous SNPs in some regions and a low density in regions of shared descent (sometimes referred to as SNP deserts; Figure S2 in Additional file 1). To detect mouse genes subject to RMAE in a genome-wide manner, we adapted an approach we had previously developed for human cells, in this case using SNP mapping arrays developed for mouse haplotype mapping [14] [GEO:GSE35678] (Figure S3 in Additional file 1). The general design and chemistry for processing of these arrays are similar to those used for the Affymetrix Human 250 K SNP Chip.

After a variety of quality control measures were implemented (Note 2 in Additional file 1) we were able to assess 69,041 SNPs. Of these, 14,458 SNPs were called as heterozygous in the genomic DNA (gDNA) of the 129S1/SvImJ × Cast/EiJ F1, and 19,587 were heterozygous in the Balb/cByJ × C57BL/6J F1. A total of 28,651 SNPs were robustly called heterozygous in the gDNA of one or both crosses (Note 2 in Additional file 1). The design of this custom array was not focused on coding polymorphisms, with only approximately 2% (585) of the 69,041 assessed SNPs falling within exons, approximately 39% (11,224) within introns, and the remainder within intergenic regions (Figure S3 in Additional file 1). As such, the experiments we performed make use of nuclear RNA, which allows for use of both intronic and exonic SNPs to detect allele-specific expression. To achieve this end, nuclear RNA extracted from immortalized cells was converted into double-stranded cDNA and used in place of gDNA in the standard Affymetrix genotyping protocol (Figure S4 in Additional file 1). In this way, we generated 'transcriptome genotypes,' which were then compared to the genotypes obtained from gDNA from the same clonal cell lines. At a particular SNP, monoallelic expression was called when the cDNA yielded a homozygous genotype call while the gDNA was called heterozygous. We considered genotyping calls from two replicate hybridizations of cDNA from each clonal cell line, as well as from non-clonal cell lines of the same F1 genotype (Note 3 in Additional file 1).

**Figure 1:**
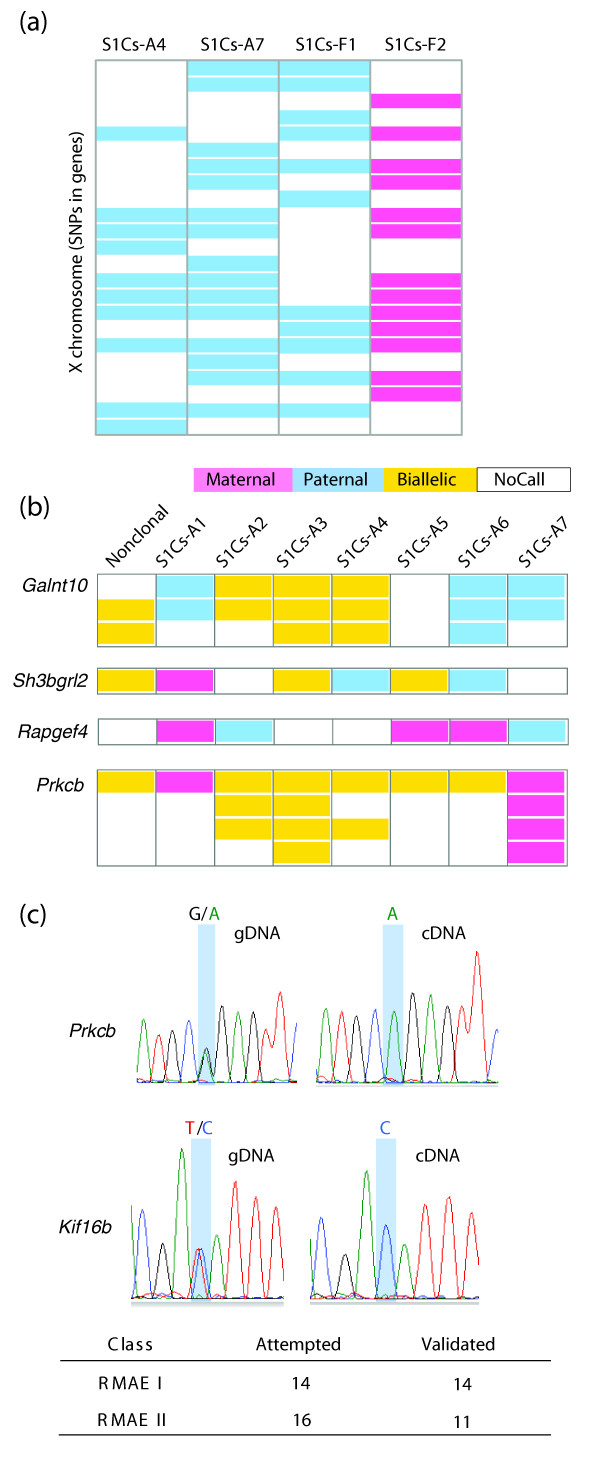
**Assessment of random monoallelic expression**. **(a) **Examples of X-chromosome inactivation in clonal cell lines from females. Each column represents an individual clone and each row represents an individual SNP within a known gene. Inset is the key for color coding. By focusing our analyses on the X-chromosome rather than autosomes, we can observe the expected chromosome-wide inactivation of one of the two X-chromosomes. **(b) **Examples of random monoallelic expression (RMAE) in autosomal genes. Colors have the same meaning as in (a). To be 'assessed,' a gene had to have either a G-score > 1 (classified as RMAE class I), equal to 1 (RMAE class II), or a G-score of 0 with 2 or more informative clones (classified as biallelic expression). See detailed explanation in Note 4 in Additional file 1**. (c) **Validation of RMAE calls with Sanger sequencing of cDNA from clonal cell lines. Comparison against the gDNA relative allelic balance is necessary to ensure that allelic imbalance seen in the nuclear cDNA did not result from PCR bias or loss of heterozygosity. The extent of allelic bias shown above (heterozygosity in the gDNA contrasted with an extreme allelic imbalance in the cDNA) is typical of RMAE genes. At the bottom is the summary of validation for randomly selected RMAE genes. Additional genes were also validated and these results, along with details on all validation experiments, are found in Table S2 in Additional file 1.

In order to confirm our assay's ability to correctly identify monoallelic expression of any type, we first examined a known example of monoallelic expression, imprinting. In order to do so, we searched specifically for autosomal genes for which monoallelic expression was observed consistently within all examined clonal and nonclonal cell populations. As expected, known imprinted genes were seen to be monoallelically expressed in our data set (Figure S5 in Additional file 1). The expressed allele is consistent with known imprinting patterns for these genes. Next we considered as a positive control a different known type of monoallelic expression, X-chromosome inactivation. Examining the monoallelic expression of X-linked genes across the length of the chromosome, we observed total agreement in the direction of X-inactivation at each queried probe (Figure 1a; Table S3 in Additional file 1). About half of the X-linked SNPs (315 of 756) were within annotated genes, while the rest were in intergenic regions. While these transcribed intergenic SNPs correctly reported X-inactivation, for all subsequent analyses we focused exclusively on genic SNPs. Consistent with the known differences in the strengths of the X-inactivation centers of the Cast/EiJ and 129S1/SvImJ strains, we observed five of six clones with the Cast/EiJ-derived X active [15]. No SNPs were present on the array that would have allowed assessment of allele-specific expression in the pseudoautosomal region of the X-chromosome, as was possible with prior studies of RMAE in humans [10].

Having completed the control experiments described above, we next examined the extent of RMAE seen for autosomal genes. To this end, we applied several filters to the data (see below and Note 3 in Additional file 1), which discarded potentially interesting observations (such as imprinting and X-chromosome inactivation), but which were essential to avoid possible technical artifacts in our search to identify random autosomal monoallelically expressed genes in the mouse. In examining the autosomes, we focused on SNPs residing anywhere within annotated genes.

For a SNP within a gene to be identified as RMAE, in addition to the presence of one or more monoallelic clones, there had to be evidence of the array's ability to call both alleles in cDNA. Additionally, the non-clonal cDNA had to be called as either biallelic or 'NoCall' (Figure 1b). Data from all contributing SNPs within a gene were considered in aggregate to determine the amount of evidence supporting that a gene is either biallelic or monoallelic. To quantify this evidence, we used a numeric 'G-score' metric (as described in [10], and Note 4 in Additional file 1). This score is weighted by the extent of agreement between multiple SNPs belonging to a given transcript in a given clone: for 99.95% of RMAE genes and 97.2% of biallelic genes this agreement is perfect (Note 5 in Additional file 1). Direct sequencing of RT-PCR products containing SNPs of interest (Figure 1c), and genotyping of RT-PCR products using primer extension on the Sequenom quantitative genotyping platform (Table S4 in Additional file 1) both served to confirm monoallelic expression. Of the class I and class II genes validated by Sequenom sequencing, between 80% and 100% of the total expression in a given gene originated from one allele.

**Figure 2:**
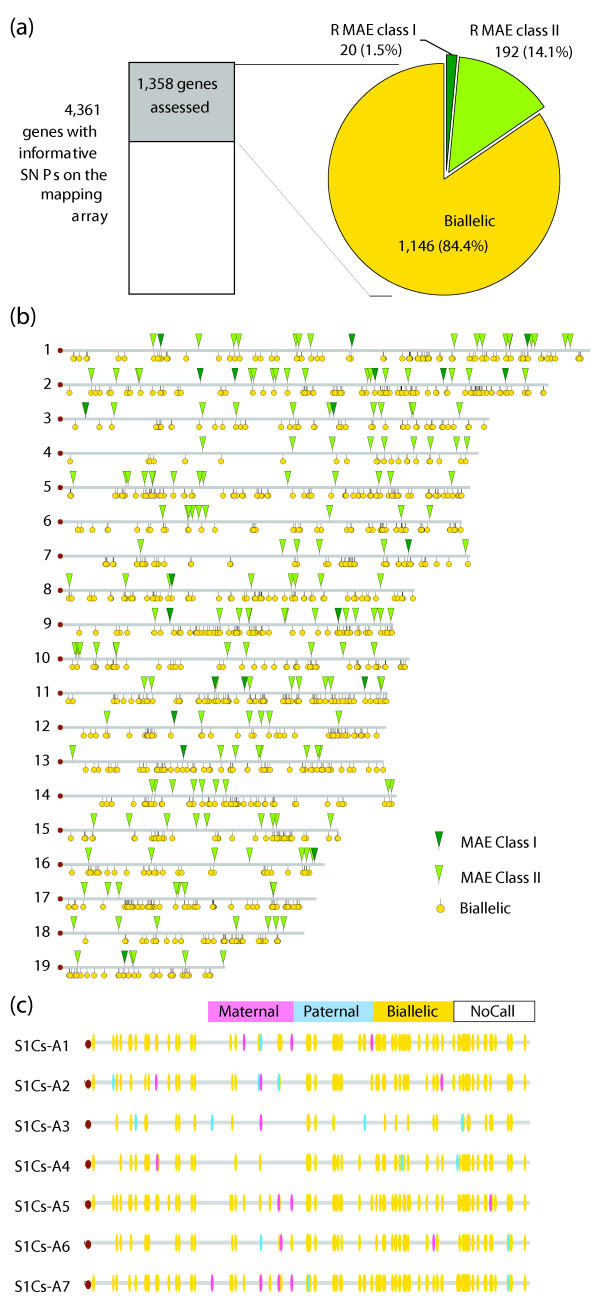
**Random monoallelic expression in the mouse genome**. **(a) **Assessed genes. Based on the identity of SNP probes present on the array and the genotypes of mice used, a total of 4,361 genes contained at least one heterozygous assessable SNP within exons or introns. The actual number assessed (shown in grey) was lower, since not all genes were expressed in the given cell type and not all SNPs passed the stringent quality control filters, which maximize specificity at the cost of sensitivity. Among these 1,358 assessed genes, 212 demonstrate random monoallelic expression (RMAE; dark green is RMAE class I, light green is RMAE class II, yellow is biallelic expression). The mean number of SNPs assessed per gene is 1.53 and the most common number of SNPs assessed per gene (mode) is 1. **(b) **Map of assessed genes on the mouse autosomes. Biallelic and RMAE genes identified in this study are located throughout the autosomes. Yellow indicates biallelic genes, light green represents class II RMAE genes and dark green indicates a class I RMAE gene. **(c) **Individual clones show unique patterns of monoallelic expression. A representative autosome, chromosome 2, is shown - all autosomes show a similar diversity of expression states. There is no coordination along the chromosome in terms of the direction of monoallelic expression. Individual clones show distinct patterns of allelic expression along the length of the chromosome, including biallelic expression.

The 28,651 queried heterozygous SNPs were further filtered for quality, requiring agreement between replicates, passing of a stringent confidence score threshold, and evidence of the ability to detect both alleles in a given assay (Notes 2 and 3 in Additional file 1). This resulted in a pool of 2,082 assessed SNPs, which reported on 1,358 genes (Additional file 3). Given the genotypes of the F1 mice studied here, a total of 4,361 genes are theoretically assessable (Figure 2a). The observation that not all 4,361 genes could be assessed was expected given that not all of these genes are expressed in the analyzed cell lines and also given the stringent confidence score thresholds assigned for accepting genotype calls (Note 2 in Additional file 1).

Of the 1,358 genes that were assessed in the lymphoblast lines, 20 (1.5%) were RMAE class I; these genes had multiple informative SNPs, and a G-score > 1. An additional 192 genes (14.1%) were called as RMAE class II; these genes have a single informative SNP per gene per clone, and have a G-score of exactly 1. Genes with a G-score between 0 and 1 were deemed inconclusive and excluded from further consideration ('RMAE class III,' Additional file 3). Genes with no evidence of monoallelic expression, a G-score of 0, and two or more clones that indicate biallelic expression (BAE), were classified as BAE genes. Thirty genes were randomly selected for validation by cDNA sequencing: 14 of 14 genes from class I and 11 of 16 genes from class II were confirmed; the remaining 5 showed BAE when sequenced (Figure 1c; Table S4 in Additional file 1). Note that the high validation rates not only confirm the accuracy of the array, but also serve to demonstrate the stability of RMAE signatures in biological replicates separated by time, as is consistent with published data [9,10].

We next examined some basic properties of the RMAE genes compared to the BAE genes. Genes that are classified as RMAE may show a nearly equal expression level of both alleles when expressed biallelically, but exhibit dramatic differences between relative allelic expression levels when expressed monoallelically (the allele appears to be either on or off, not simply dampened in expression level). This is exemplified in Figure 1c and has been quantified by Sequenom transcriptome genotyping. The genes from RMAE classes I and II encode gene products with a variety of functions and expression patterns (Table 1), which is also true of the BAE genes. In contrast to the human results, we did not find any statistically significant Gene Ontology term enrichment within the set of mouse RMAE genes. Finally, RMAE genes are scattered throughout the mouse genome (Figure 2b), with monoallelic and biallelic genes interspersed, which is similar to the distribution seen in the human genome. Along with the interspersion of RMAE and BAE genes, each clonal cell line we analyzed appears to also have a unique configuration of allele-specific states (Figure 2c). Among the clones, the same gene may be expressed from either allele or from both alleles (Figure 1b); note that in this analysis, and throughout this work, we refer to the alleles as maternal or paternal not to imply an imprinting mechanism, but rather to distinguish between the two alleles. Within a given clone, different monoallelic genes show maternal or paternal expression apparently independently (Figure 2c). Thus, RMAE leads to a significant level of epigenetic heterogeneity between clonal cell lines that are otherwise identical.

**Table 1:** Diverse functions of random monoallelic expression genes

Gene	Class	Full name	Prominent molecular function(s)	Most highly expressed in
*Atxn1*	RMAE I	Ataxin 1	RNA binding	Brain tissues, immune cells
*Bcl2*	RMAE I	B-cell leukemia/lymphoma 2	Protease binding/transcription activator	Blood progenitor cells
*Inpp4a*	RMAE I	Inositol polyphosphate-4-phosphatase, type I	Phosphatase	Testes
*Myh10*	RMAE I	Myosin, heavy polypeptide 10, non-muscle	Motor activity	Many
*Neb*	RMAE I	Nebulin	Actin binding	Skeletal muscle
*Rasgrp1*	RMAE I	RAS guanyl releasing protein 1	Nucleotide exchange factor, ion binding	Brain tissues, immune cells
*Slamf6*	RMAE I	SLAM family member 6	Receptor activity	Immune cells
*Zdhhc2*	RMAE I	Zinc finger, DHHC domain containing 2	Ion binding, transferase activity	Bone marrow, brain tissues
*Atp2a2*	RMAE II	ATPase, Ca++ transporting, cardiac muscle, slow twitch 2	Calcium binding/ATPase activity	Heart
*Bmpr1a*	RMAE II	Bone morphogenetic protein receptor, type 1A	Many	Many
*Cad*	RMAE II	Carbonyl-phosphate synthetase 2, aspartate transcarbamylase, and dihydroorotase	Enzymatic activity	Embryonic stem cells
*Creb1*	RMAE II	cAMP responsive element binding protein 1	DNA binding/transcription factor binding	Many
*Cul1*	RMAE II	Cullin 1	Ubiquitination	Many
*Dock9*	RMAE II	Dedicator of cytokinesis 9	Guanyl-nucleotide exchange	Many
*Esr1*	RMAE II	Estrogen receptor 1 (alpha)	Binding factor	Uterus, ovary, pituitary
*Gart*	RMAE II	Phosphoribosylglycinamide formyltransferase	Nuclease binding/transferase activity	Stem cell, blood progenitor cells
*Igf2bp3*	RMAE II	Insulin-like growth factor 2 mRNA binding protein 3	Nucleotide binding/mRNA binding	Embryo, placenta, osteoblasts
*Kif1c*	RMAE II	Kinesin family member 1C	Nucleotide binding/microtubule motor	Many
*Lrp11*	RMAE II	Low density lipoprotein receptor-related protein 11	Receptor activity	Pituitary, neuronal
*Lyplal1*	RMAE II	Lysophospholipase-like 1	Hydrolase activity	Adipose
*Mapk8*	RMAE II	Mitogen-activated protein kinase 8	Nucleotide binding/kinase activity	Many
*Mtap2*	RMAE II	Microtubule-associated protein 2	Microtubule/neuronal structure	Brain tissues
*Myb*	RMAE II	Myeloblastosis oncogene	DNA binding/transcription regulation	Blood progenitor

Listed for a subset of RMAE genes is the gene name, the primary tissue(s) of expression and the gene function(s).

We next asked if there was a correlation in the direction of expression (maternal allele or paternal allele) between neighboring monoallelic genes along a chromosome within the same clonal cell line. From visual inspection it is apparent that autosomal RMAE does not have the strict chromosome-wide coordination of the X-chromosome inactivation (Figure 2c). Nevertheless, to explore the possibility of a more subtle coordination between neighboring genes, we calculated the minimal distances between neighboring RMAE genes and defined their relative allelic states, giving a label of 'agree' when both express the maternal allele or both express the paternal allele, and 'disagree' if they express opposite alleles. In the seven lymphoblast clones of the 129S1/SvImJ × Cast/EiJ genotype, 85 neighboring gene pairs agreed and 78 pairs disagreed. The observed counts (85 and 78) are roughly equal (chi-square *P *= 0.58), consistent with the notion that the choice of the active allele is independent for each RMAE gene. A further analysis examining only the RMAE gene pairs within 5 Mb yielded similar results, with 14 gene pairs that agreed and 11 pairs that disagreed (chi-square *P *= 0.55). Note also that, for both the general analysis and the one focused on pairs within 5 Mb, the mean of the distances for those pairs that agreed was similar to the mean distances for those pairs that disagreed (30.1 Mb versus 25.7 Mb, and 2.1 Mb versus 1.7 Mb, respectively). While it is possible that there is coordination at extremely close distances that fall below the resolution of this analysis, our findings are consistent with the notion that the choice of an active allele at each RMAE locus is made independently of the adjacent loci. Finally, we saw no significant relationship between the amount of RMAE observed and the gender of the mouse from which the clonal lines were derived (details in Note 6 of Additional file 1).

**Figure 3:**
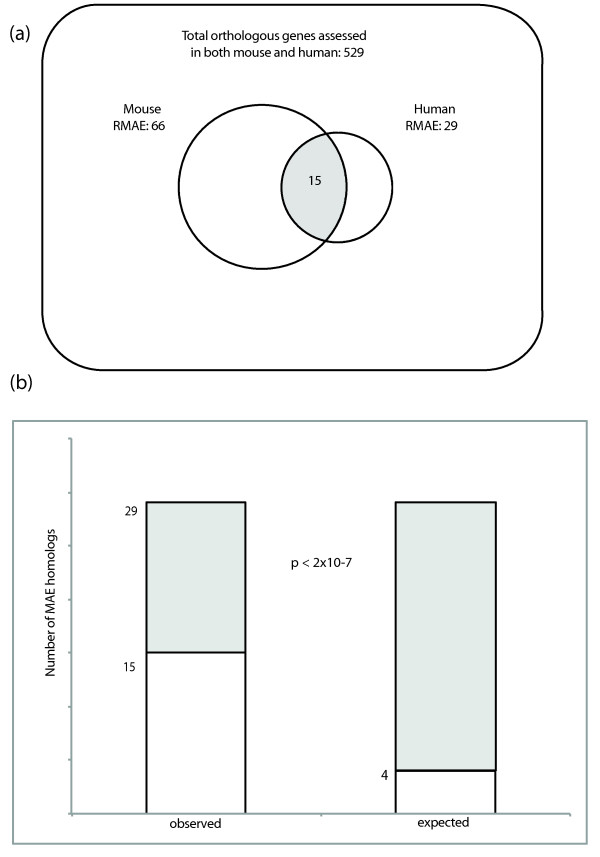
**Comparison of random monoallelic expression in human and mouse genomes**. **(a) **Human and mouse random monoallelic expression (RMAE). Of all orthologous gene pairs between mouse and human, there were 529 for which the gene was assessed (as either biallelic expression, RMAE class I or RMAE class II) in each organism. Of these orthologs, 66 were RMAE in mouse and 29 were RMAE in human, resulting in a subset of 15 genes that were assessed as RMAE in both organisms. **(b) **The number of assessed orthologs showing RMAE in both species is greater than would be expected by chance (Note 6 in Additional file 1). The observed and expected overlaps are shown as subsets of the gray bars, which represent the maximum possible overlap, as defined by the total number of RMAE genes (29) observed in human (for an overlap of 15 or more genes; hypergeometric *P *< 2 × 10^-7^).

With both mouse and humans exhibiting monoallelic expression in a subset of genes (15.6% in mouse and 9.5% in human), we next examined the extent to which genes that exhibit RMAE in one species also display RMAE in the other. To assess the extent of this overlap, we focused on mouse genes with exactly one human ortholog and for which a call (either RMAE class I, RMAE class II or BAE) was made in both mouse and human data. The maximum possible amount of overlap is the total number of RMAE orthologous genes in the species with fewer such genes (29 human RMAE genes). A simple model would suggest that the overlap would be no more than that predicted by random chance; thus, we multiplied the proportion of orthologs that demonstrate RMAE in each species by the total number of assessed orthologs (this model assumes *a priori *that any ortholog is equally capable of showing RMAE). The observed data show much higher overlap between mouse and human RMAE than would be expected by chance: approximately 4 (3.6) expected, 15 observed (22.7% of total mouse RMAE), and hypergeometric *P *< 2 × 10^-7 ^(Figures 3a, b). This excess overlap suggests conservation for the potential to exhibit random monoallelic expression.

Finally, we explored whether or not there was a form of skewing in the allelic choice for RMAE genes. This type of skewing would be analogous to the primary X-inactivation, wherein one chromosome is more frequently active than the other. This phenomenon has been well characterized in mice [15,16] and there are some examples in human. In the case of autosomal RMAE, however, in light of our analysis above, the skew would be observed at the level of individual genes rather than the whole chromosome. Essentially, we were asking if, for a subset of RMAE genes, instead of a 50:50 split, one of the two alleles is preferred.

Given the number of clones examined, it is difficult to assess this kind of skewing at an individual gene level. However, when examining the entire set of our RMAE genes reported here, it becomes apparent that a bias in the choice of which allele is expressed occurs more frequently within these data than can be explained by chance. Furthermore, this bias cannot be explained by uniformly skewed types of expression (such as the skewing found due to imprinting or *cis*-regulatory variation) as we have in place filters that remove such expression profiles from consideration (Note 3 in Additional file 1). It would seem for some genes, therefore, that the two alleles are somehow unequal in their ability to be monoallelically expressed.

**Figure 4:**
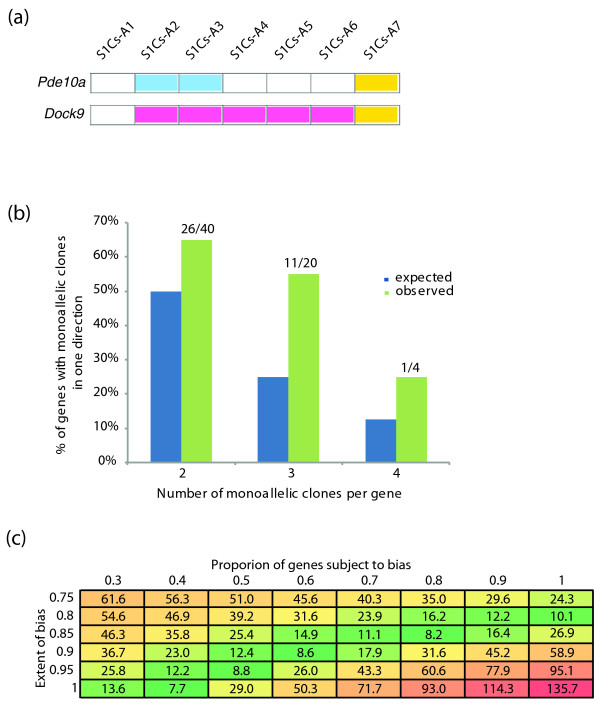
**Skewed monoallelic expression in the mouse**. **(a) **Examples of genes with apparent skewed monoallelic expression. Same display conventions as Figure 1a. For a subset of random monoallelic expression (RMAE) genes, such as those shown here, we only observed RMAE in one direction (either monoallelic maternal or monoallelic paternal). **(b) **Skewed genes. For any given gene, the number of clones is too low to make an observation of skewed RMAE significant. When considering the genome-wide data, however, it becomes apparent that an observation of bias in the direction of RMAE occurs more often than can be explained by chance. When examining genes that show RMAE in only one direction, we can compare the number of genes observed (green) to that which would be expected by chance (blue) for genes with two, three or four monoallelic clones. In each case, the number of genes with RMAE clones solely in one direction is higher than expected (two clones, *P *= 1.92 × 10^-2^; three clones, *P *= 9 × 10^-4^). See main text and Note 6 in Additional file 1 for details. **(c) **The observed skewed RMAE is consistent with a range of simple models. By varying the percentage of genes subject to skewed RMAE, and by varying the probability of seeing one allele rather than the other allele for those genes with skewed RMAE, we estimated how closely simple models approximate the observed numbers of genes with monoallelic clones all in one direction. Shown in each cell is the sum of squares of differences between the observed and expected number of genes with two, three, and four clones all in one direction; the smaller the value is, the more closely the model approximates the actual observed values.

Evidence for this type of skewing can be found by examining cases in which all the clones displaying monoallelic expression of a given gene express the same allele (examples shown in Figure 4a). Among the genes for which a given number of clones display monoallelic expression (two, three or four clones), we counted the genes for which every clone displaying monoallelic expression expressed the same allele. We then compared these observed numbers to the numbers that would be expected by chance; genes with two, three or four clones with monoallelic expression would be expected to show frequencies of 0.5, 0.25 or 0.125, respectively, given an equal probability of being chosen for each of the two alleles (Figure 4b). For each set of genes with a given number of clones, we observe greater than expected numbers of genes displaying expression solely from one allele (*P *< 0.05; Note 7 in Additional file 1). The direction of skewing (maternal versus paternal) varies from gene to gene. These results are consistent with a range of models, including one in which 40% of genes have a near complete bias for one of the alleles to be preferentially expressed as well as a model in which 80% of the genes choose one allele 85% of the time, with the other allele chosen for RMAE 15% (Figure 4c; Note 7 in Additional file 1).

## Discussion

RMAE is of interest as an epigenetic phenomenon as it requires unequal regulation of two alleles, even if they are identical. Moreover, since parental imprinting mechanisms are not at play, there is a requirement for the RMAE of individual genes to be established independently in individual cells during development, much like X-inactivation in females, except that the decision occurs at the level of the individual autosomal gene as opposed to the entire X-chromosome. DNA rearrangement-mediated RMAE of immunoglobulin and T-cell receptor genes were the only known examples of autosomal RMAE until the report of monoallelic expression in the odorant receptor gene family and subsequent reports of a handful of other genes, most involved in the immune and the immune chemosensory systems.

Using SNP genotyping arrays capable of genome-scale query, we have recently established that RMAE of autosomal genes is widespread in the human genome, affecting approximately 10% of assessed genes [10]. In order to understand the extent of this phenomenon and its evolutionary conservation, in this work we examined RMAE in mouse cells, using a genome-scale approach in clonal cell lines. We observe widespread monoallelic expression in the mouse, comprising over 10% of genes, as evidenced by allele-specific expression observed in one or more mouse lymphoblast clonal cell lines. A smaller but still sizeable number of genes were found to be RMAE in fibroblasts (Figure S1 and Note 1 in Additional file 1). As we found in analyzing human genes, for most RMAE mouse genes, the allelic expression state varies from clone to clone: sometimes maternal monoallelic, sometimes paternal monoallelic, sometimes biallelic (98.1% of genes have at least one BAE clone). We did not repeat the extensive *in vivo *validation of RMAE that was performed for the earlier study of RMAE of human genes. As such, it is formally possible some of the RMAE observed in mouse cell lines differs from the expression patterns *in vivo*. However, given the conclusiveness with which RMAE detected in human cell lines was validated *in vivo *taken together with the overall similarity of gene regulation mechanisms in human and mouse, it is reasonable to expect that the RMAE found in mouse cell lines generally reflects the situation *in vivo*.

As for humans, the choice of active allele is not coordinated chromosome-wide; in a given clone, the expression state of each monoallelic gene is independent of the others. As a result, each clone has a unique signature of allele-specific expression, creating extensive epigenetic heterogeneity in otherwise identical cells. The diversity of patterns observed within a population of cell lines could be explained by an initial period of plasticity (or random choice) followed by a fixation of each allele's allelic expression state. The properties of RMAE described above for the mouse are similar to what was observed in human clonal lymphocytes [10].

Autosomal RMAE has the potential to impact biological function by creating three distinct cell states for each gene in instances when both alleles encode functional gene products. For each given gene, these states would be defined by expression of the maternal allele, the paternal allele, or both alleles. The observed stability of the allele-specific choice in a given clone [9,10], together with *in vivo *clonal expansion, can lead to growth of macroscopic patches of tissue with subtly distinct properties. In studies of human RMAE, such patches were shown in the normal placenta [10]. In general, the size of these patches would be dependent on the stage in development at which the allelic choice is made for each developing tissue. Given the large number of autosomal genes involved, there is a clear potential for RMAE to contribute to phenotypic differences among individual organisms.

Considering orthologous genes assessed for RMAE in similar cell types in mouse and human, we find that the number of genes subject to RMAE in both species is five-fold greater than would be expected by chance (Figures 3a, b). We can thus conclude that regulatory features allowing a gene to be RMAE were present in the last common ancestor of rodents and primates, and that these features have been maintained in the intervening 65 to 85 million years [17]. Under one interpretation, such evolutionary conservation could be due to the selective advantage of these genes' RMAE. Indeed, for some previously known examples of autosomal monoallelic expression, such as olfactory receptors and immunoglobulins, the adaptive advantage of monoallelic expression is clear: it confers a unique specificity to otherwise identical cells. However, it is also formally possible that for at least some of the newly identified mouse RMAE genes, RMAE is not in itself adaptive, but is rather a consequence of other regulatory features being acted upon by selective pressures. Another possibility supported by these data would be that RMAE of some genes adversely affects the fitness of the organism to the point that these genes are excluded from RMAE, resulting in obligate, and thus conserved, BAE. This would thus limit the pool of orthologs that have the potential to display RMAE.

In a striking difference from human RMAE, in 129S1/SvImJ × Cast/EiJ murine cells we often observe statistically significant 'skewed RMAE', wherein, for a given gene, there is preferential expression of one allele for cells with monoallelic expression (Figure 4). In extreme cases that still lie within the boundaries of our definition of RMAE, all instances of monoallelic expression originate from the same allele, while the other allele is active only in clones that show BAE. This skewed RMAE resembles the skewed X-chromosome inactivation that has been observed in mouse F1 hybrids, and that has been traced to distinct properties of the sequence elements known as X-inactivation centers [15]. In the case of skewed X-chromosome inactivation, each cell still has chosen one or the other copy of the X-chromosome to be active. What is skewed is the relative abundance of these cells with one X active versus cells with the other X active.

**Figure 5:**
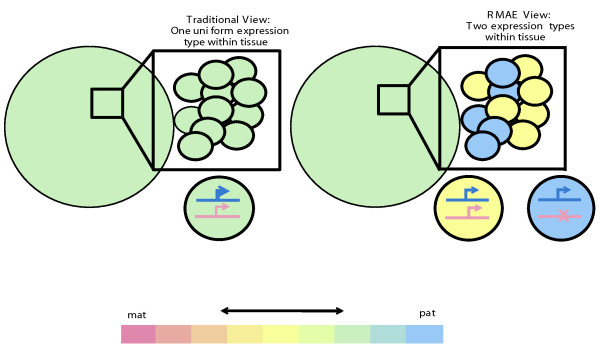
**Clone-specific monoallelic expression and tissue-scale allelic imbalance**. Allelic imbalance has been noted for a variety of genes in different tissues and is typically attributed to either parent-of-origin imprinting or to *cis*-regulatory variants. Skewed random monoallelic expression (RMAE), when one allele is preferentially chosen for expression, could also result in a tissue-wide allelic imbalance. In a traditional view (left) the expression level varies between the two alleles, with the paternal (pat) allele contributing more. This is uniformly true among the cells in the tissue. By contrast, in the scenario depicted at right, a difference in the relative abundance of cells with different RMAE states results in a tissue-scale allelic imbalance. Mat, maternal; Pat, paternal.

The notion of skewed RMAE has important implications for the interpretation of data measuring allele-specific expression of autosomal genes, since such skewing could underlie some instances in which 'incomplete imprinting' has been observed, as well as instances where allelic imbalance tracks with strain of origin (and *cis*-regulatory polymorphisms have been presumed to be the sole regulatory mechanism). For example, a gene that is equally expressed from both alleles in half of the cells, and from only one allele in the other half of the cells, would appear to show significant allelic imbalance when assessed in a mixed cell population (for example, 2:1 if the level of expression is fixed per allele; Figure 5). However, the underlying mechanisms and functional consequences (especially for genes with cell autonomous functions, such as tumor suppressors) would be quite different than in a 'classical' allelic imbalance (that is, one due to the interaction of *cis*- and *trans*-factors [18].

Finally, we compared the RMAE seen in immortalized lymphoblasts to that seen in fibroblast lines. Similar to results reported for lymphoblasts, fibroblasts demonstrate RMAE across a large number of genes throughout the genome (Figure S1 in Additional file 1). By contrast, a lower overall level of RMAE (Note 1 in Additional file 1) was seen in fibroblasts, a finding that is consistent with the idea that RMAE is cell type specific [9,10].

## Conclusions

Autosomal RMAE is widespread in the mouse, and observed for many of the same genes as were found to be RMAE in human. The genes subject to RMAE in the mouse are scattered throughout the genome and have a wide variety of functions and expression patterns. RMAE is observed in two cell types and two different mouse genotypes. Intriguingly, there is a trend for certain genes to be observed in only one of two possible monoallelic expression states, arguing for an imbalance in the probability of expression for the two alleles.

The mechanisms allowing differential expression of the active and inactive alleles of most RMAE genes remain to be explored, as do the DNA sequences that may control RMAE. We expect that there are a number of mechanisms involved, especially considering the large number of RMAE genes, their wide distribution across the genome and their widely varying functions. Moreover, for skewed RMAE, either sequence polymorphisms or imprinting must underlie the two alleles of some genes having differential expression probabilities.

Unraveling the mechanisms of RMAE has the potential for uncovering new mechanisms of gene regulation. Already many mechanistic insights have emerged from studies of the first known autosomal RMAE genes: the extraordinarily diversifiable immunoglobulin and T-cell receptor genes. Previously published studies using human lymphoblastoid clonal lines, native T cells and keratinocytes have shown that ablation of methylation achieved by treatment with either 5-azadeoxycytidine or 5-aza-2'deoxycitidine lessens the allelic expression imbalance observed in five confirmed RMAE genes [19,20].

Beyond the mechanisms involved in regulation of different RMAE genes, understanding the extent to which RMAE leads to phenotypic differences between individuals could be of use in understanding both Mendelian and complex genetic diseases. Even in Mendelian disorders, there is often phenotypic variability within families that can be reasonably ascribed to differences in genetic background or to gene-environment interactions. Autosomal RMAE provides another potential explanation for phenotypic variability, similar to the variability in phenotype observed in females who carry certain mutations on the X-chromosome. In complex genetic diseases where many genes contribute to the genetic predisposition, again autosomal RMAE has the potential to explain some of the variability in phenotype.

## Materials and methods

### Source material

As a source of cells, F1 mice were used from each of the following mouse crosses (female × male): (a) 129S1/SvImJ × Cast/EiJ; (b) 129S1/SvImJ-Y11 × Cast/EiJ; (c) Balb/cByJ × C57BL/6J; (d) C57BL/6J × Balb/cByJ. The use of these different F1 crosses allowed for both in-depth examination of SNP dense regions as well as a comprehensive overall view of the chromosomes (Figure S2 in Additional file 1); 129S1/SvImJ and Cast/EiJ have very different breeding lineages and so are polymorphic at many areas throughout the genome. By contrast, Balb/cByJ and C57BL/6J have a more similar breeding history, and so areas of shared descent are extremely low in polymorphisms ('SNP deserts') while areas not of shared descent are extremely high in polymorphisms ('SNP jungles'). 129S1/SvImJ-Y11 mice, which were used in some breedings (Table S1 in Additional file 1) are identical to 129S1/SvImJ aside from the insertion of a transgene on chromosome 2; this transgene has no influence on our results and was inserted for the purposes of an unrelated study.

Primary cells from individual F1 embryos were gathered from either E14 embryonic liver (pre-B cells) or adult ear tissue (fibroblasts). Cells were immortalized using standard procedures for infection with Abelson murine leukemia virus [21] for the pre-B cells or transformation with SV-40 large T antigen [10] for the fibroblasts. We used a fluorescence-activated cell sorter to place single cells in 96-well plates; wells with growth were passaged to establish monoclonal cell lines. Only one clonal line was established per tissue type per mouse, ensuring the independence of clonal samples. Primary cells were passaged 15 times during the process of immortalization and prior to sorting. After single cells were used to establish subclones, cells were passaged 30 times to grow to sufficient cell number prior to RNA harvesting. Cells used for validation experiments were either from the original sample (for Sequenom validation) or from populations that had undergone an additional 5 to 15 passages (for Sanger sequencing based validation). Immortalized polyclonal lines were also established from primary samples of both pre-B lymphoblasts and fibroblasts by the same methods, excluding the single cell sorting.

### Experimental preparation

Both nuclear RNA and gDNA were extracted from each cell line analyzed. For the expression assay, nuclei were isolated from cell lines and RNA was extracted from these nuclei using Trizol and standard procedures, including DNase treatment (Ambion Turbo DNAfree, Austin, Texas, USA). Single-stranded cDNA was created in a random-primed reverse transcription reaction and subjected to second strand synthesis (Invitrogen SuperScript II, Carlsbad, California, USA). gDNA was extracted using QIAamp DNA Blood Mini Kits (Qiagen, Hilden Germany). Both gDNA and cDNA were independently processed (including NspI restriction endonuclease digestion) and hybridized to custom-manufactured mouse SNP genotyping arrays [14], which are designed similarly to the Affymetrix Human 250 K SNP genotyping chip. The complete set of primary data, including genotype calls for all arrays, SNP annotation and associated files, has been deposited in NCBI's Gene Expression Omnibus (Zwemer *et al*., 2012) and is accessible through series accession number [GEO:GSE35678].

Parental genotypes used were published data that are publically available, much of which was previously confirmed using the same SNP chip we used (Note 2 in Additional file 1) [14].

### Data processing

Array signals were processed using the Dynamic Model Mapping Algorithm (DMMA; Note 2 in Additional file 1). For each clonal line, two replicate arrays were hybridized for each cDNA sample and one array per gDNA sample (for a total of three arrays per clonal line). Once calls had been generated using DMMA, data were processed using a collection of custom software and filters, collectively referred to as 'MAEstro,' which were designed to identify and rank RMAE genes. In the following explanations, the term 'analysis set' refers to the collection of DMMA-produced data submitted as a group for MAEstro analysis. Due to the nature of the filters applied, the results vary based on the composition of the analysis set (Note 3 in Additional file 1). Within each MAEstro analysis set are data associated with exactly one non-clonal line and at least two clonal lines. For both clonal and non-clonal lines, MAEstro required DMMA calls from one gDNA array and two technical replicate cDNA arrays.

More detailed information about MAEstro analyses is available on request. In short, the 'transcriptome-derived genotype' (based on two technical replicate cDNA arrays) is compared to the gDNA-derived genotype at each SNP on the array, for each clonal and non-clonal line examined and the filters described in Note 3 in Additional file 1 are applied. Array probes corresponding to loci not located in known transcripts were excluded. Use of DMMA was chosen over a BRLMM (Bayesian Robust Linear Model with Mahalanobis distance) type method because DMMA examines arrays individually, rather than forcing artificial normalization among arrays. Forcing a BRLMM normalization would destroy signatures of RMAE; therefore, methods of genotype calling that require BRLMM normalization are not suited to RMAE screens. DMMA has the further advantage that it uses the array's mismatch probes to facilitate accurate allele-specific genotype calling.

## Abbreviations

BAE: biallelic expression; BRLMM: Bayesian Robust Linear Model with Mahalanobis distance; DMMA: Dynamic Model Mapping Algorithm; gDNA: genomic DNA; RMAE: random monoallelic expression; SNP: single nucleotide polymorphism.

## Competing interests

The authors declare that they have no competing interests.

## Authors' contributions

AJC designed the experiments, analyzed the data and drafted the manuscript. AAG and LMZ designed the experiments, performed the experiments, analyzed the data and drafted the manuscript. BT and AZ assisted with data analysis pipeline development. MD designed the mouse array. AK provided the annotation for array probe sets and designed the mouse array. All authors read and approved the final manuscript for publication.

## Supplementary Material

Additional file 1 **Supplementary Notes, Figures and Tables**. These include methodological notes, additional data Figures S1 to S5 and Tables S1, and S3 to S4 [22]. Click here for file

Additional file 2 **Table S2 - complete allele-specific expression information for fibroblast cell lines**. Click here for file

Additional file 3 **Table S5 - complete allele-specific expression information for lymphoblastoid cell lines**. Click here for file
