# Supplementary material for: Autosomal monoallelic expression in the mouse
Source: Genome Biol. 2012 Feb 20;13(2):R10. doi: 10.1186/gb-2012-13-2-r10 (PMC3334567; doi:10.1186/gb-2012-13-2-r10)
Supplement: Additional file 1 — Supplementary Notes, Figures and Tables. These include methodological notes, additional data Figures S1 to S5 and Tables S1, and S3 to S4 [22]. [file gb-2012-13-2-r10-S1.PDF]

Supplementary Notes, Figures and Tables for Zwemer et al. 2012

Note 1. Fibroblasts

Fibroblast and lymphoblast cell lines were analyzed separately to ensure that the stringent filters described in Supplementary Note 3 are appropriately applied to each cell line. For example, evidence that a gene is not imprinted in fibroblasts is insufficient to exclude a conclusion of imprinting from lymphoblast results, and this exclusion is a necessary step to correctly identifying genes that are randomly monoallelically-expressed.

Because of the random nature of RMAE, a gene may be expressed as biallelic in some clones and monoallelic in others (note that the expression pattern is stable within a given clone). The list of genes that display RMAE is thus a result of considering expression states from multiple clonal lines, only some of which would be expected to exhibit RMAE for each given gene. It follows that as we examine more clones, there are more opportunities to observe genes in a monoallelic state. Therefore, to compare the amounts of RMAE in two different tissues we must consider the same number of clones from each tissue type. As such, we consider two clonal fibroblast lines with one nonclonal fibroblast line, and two clonal lymphoblast lines with one nonclonal lymphoblast line. We found that fibroblasts show lower levels of RMAE than those seen in lymphoblast lines (Figure S1).

The various types of monoallelic expression are often tissue-specific: some imprinted genes are tissue-specific [1]; X-chromosome inactivation is imprinted in the mouse extraembryonic tissues, but random in the embryo proper [2]; p120 Catenin (*Ctnnd1*) is RMAE in mouse B cells, but not in fibroblasts [9]. The same appears to be the case for widespread RMAE in the mouse: we observed a greater fraction of genes subject to RMAE in transformed lymphoblasts than in fibroblasts (Figure S1). For any given individual gene, lack of agreement between the cell types (i.e. RMAE in one type, but BAE in the other) could be a reflection of the underlying biology. It could also, however, be due to the limited number of independent clones we analyzed, as by definition an RMAE gene will not be seen as monoallelic in every analyzed clone, or even the first few examined. When controlling for the number of clones examined, however, the lower total number of RMAE genes seen in the fibroblast lines suggest that the extent of RMAE is lower in fibroblasts.

Note 2. Probe Selection

Probes originally selected for use in the array correspond to loci known to be polymorphic among a variety of inbred and inbred-outbred mouse strains. These include Oxford SNPs and Perlegen SNPs [14], all of which are publically available for each of the strains used in this work. Several lines of logic were used in selecting the probes for use in our study from among the 149,116 probes present on the custom mouse genotyping array. A first round of stringent data analysis of the custom mouse array was made during other studies using this array [14] and resulted in identification of many probes that were thought to be faulty, insufficiently specific, or unreliable. Following the example of Kirby et al. [14], we removed these underperforming probes from our own analysis. Additionally, this custom array is intended for use with a variety of mouse strains, and thus not all the probe sets present on the array correspond to polymorphisms that exist among the strains we used. Next we marked probes that correspond to multiple overlapping transcripts, whether both are sense or one is

antisense. Results for these probes are considered separately and not discussed in the main text of the paper. Rather, they can be found in Tables S2 (Additional file 2) and S5 (Additional file 3). To focus on only the SNPs of interest, we further selected a subset of probes which were not only theoretically polymorphic within our F1 crosses, but which also successfully and reliably called that polymorphism in the gDNA of the majority of our clones, using the default DMMA confidence score cut-off of 0.325. So as to be conservative, any probe that was called homozygous for the gDNA of a heterozygous clonal line at a known polymorphic site was discarded from all analyses for all lines. Reciprocally, any probe that was called heterozygous in the published parental gDNA (known to be homozygous) was excluded [14]. Finally, a confidence score (CS) cut-off was established for autosomal loci at a DMMA confidence score of 0.1; a confidence score cut-off of 0.15 was chosen for X-chromosome probes. Probe calls with CS >= the cut-off received a designation of NoCall. A NoCall means that the best fit model is insufficiently better than the next best model (as described in the Affymetrix Human 500K mapping array manual). This may be due to an absence of signal (no or low expression) or it may be due to an inability to conclusively match the data to one genotype even when detected expression is relatively high. The confidence score cut-offs were determined empirically, based on the trade-off between replicate array agreement and number of assessed SNPs. This CS threshold must be passed by both replicates of cDNA in order to avoid a NoCall at a given locus. While stringent, this set of thresholds and filters was implemented because we would prefer false negatives to false positives in the search for RMAE genes.

Note 3. MAEstro Filters

In order to focus specifically on RMAE, MAEstro implemented several filters, each of which acted on the level of individual probe sets (corresponding to individual SNPs). First, only probes corresponding to autosomal loci were considered (except in the case of an assay confirming X-chromosome inactivation, in which case only the X-chromosome probes were examined (Fig. 1a). Second, when considering the data from all clonal and nonclonal lines within an analysis set (i.e. the array data submitted for analysis as a group), for each probe set there had to be evidence of the ability to accurately detect each allele of the SNP, either by making a biallelic call in at least one sample or by making both possible monoallelic calls (i.e. both monoallelic-paternal and monoallelic-maternal). Third, a given SNP was excluded if it was called as homozygous in the cDNA of the nonclonal population. The polyclonal lines are, by definition, comprised of multiple distinct clonal lineages, each with its own unique signature of monoallelic expression. Thus, the aggregation of signatures from constituent clones is expected to result in a biallelic signature (much as a multiclonal patch of tissue in a mammalian female will appear to have biallelic X-chromosome expression). By contrast, genes that display monoallelic expression in the heterogeneous cell population of a polyclonal line are likely candidates for either strong *cis*- or imprinted-type regulation, and so should be considered separately from genes showing an RMAE type signature. By removing from consideration any such genes that show homozygous cDNA calls in the nonclonal line we narrow our focus only to genes that show RMAE patterns.

Disregarding SNPs that show a homozygous call in the nonclonal cDNA also serves a secondary purpose of limiting the confounding effects of cross-hybridization (a consideration for any array-based experiment). For example, if cross hybridization were an issue, it would be an issue consistently across all samples (clonal and nonclonal alike). Therefore, the fact that our assay relied on the detection of variation within these

samples argues against the possibility of false positives for RMAE due to cross hybridization.

Note 4. G-score

A metric was used to assess the extent to which a given gene displays evidence of RMAE (a “G-score”). This metric, originally devised for use in the screen for RMAE in humans [10], has several constituent parts. For each gene the number of biallelic SNPs in each clone and the number of monoallelic SNPs in each clone are counted and summed. These numbers are each averaged by the numbers of informative biallelic clones or monoallelic clones to give the values D_B and D_M, respectively (see below). In cases in which no biallelic clones are observed, D_B has a default value of 1. A clone in which two or more SNPs within a gene disagree on the call is considered a “conflict clone” (C) and as such is not counted toward either the monoallelic or the biallelic totals. By design, this metric is conservative, penalizing a gene heavily for clones that give conflicting information.

$$G = \frac{{D_M}^2}{D_B \times (C+1)}$$

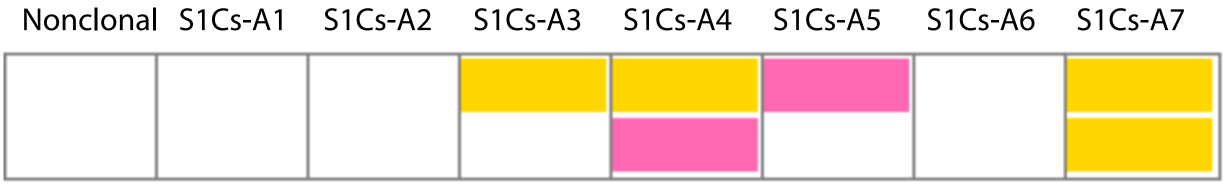

In this example, *Elf2*, columns show results for each clone while rows show results at each SNP. Pink indicates monoallelic maternal expression and yellow indicates biallelic expression. S1Cs-A7 contributes two biallelic SNPs and S1Cs-A3 contributes 1, for a total of 3: D_B=3/2=1.5. S1Cs-A6 contributes one monoallelic SNP: D_M=1. Clone S1Cs-A4 is a conflict clone; as such it contributes neither to D_B nor to D_M, but causes a lowering of the G-score: C=1; G=0.33.

If a gene was informed by SNPs in more than one F1 cross, the gene was characterized by the highest G-score observed (“G_{max}”). For example, the RMAE gene *Slamf6* was reported as RMAE based on data from two types of F1 mice. In Balb/cByJ x C57BL/6J a G-score of 3.0 was reported, while in 129S1/SvImJ x Cast/EiJ a G-score of 1.0 was reported. Thus the G_{max} for this gene, as reported in ST4, is 3.0.

Note 5. Conflict SNPs

In the case where multiple SNPs within a gene disagree on the expression profile (biallelic versus monoallelic maternal versus monoallelic paternal) the clone was designated a conflict clone for that gene. This negatively impacted the G-score, reflecting the diminished confidence we had in these data and thus ensuring that

potentially faulty probes do not dominate our results. In reality, very few genes contain SNPs conflicting with one another (only one monoallelic gene and less than 2.5% of biallelic genes).

Note 6. RMAE and Gender

Comparisons of male- and female- derived clonal lines yielded comparable amounts of RMAE within each analysis set. Using 3 clonal and 1 nonclonal line as inputs, the amount of RMAE seen for males is 6.5% and the amount seen for females is 6.2% of assessed genes.

Note 7. Statistics

In Figure 3 we compare expected and observed numbers of genes that are RMAE for both mouse and human. To calculate the expected number, we considered genes that have orthologs in both human and mouse and which have been observed to be either RMAEI, RMAEII, or BAE in both mouse and human studies ("assessed orthologs"). Of these 529 assessed orthologs, the expected number to be RMAE in both is the %RMAE in the human subset x %RMAE in the mouse subset x total number of genes ($29/529*66/529*529 \approx 3.6$). The maximum possible number of shared RMAE genes is 29 since the number of genes displaying overlap cannot be more than the total number of genes in the species showing fewer genes subject to random monoallelic expression.

In Figure 4b we compare expected and observed amounts of skewing. The expected values were created by using a simple coin-flip experiment. If we consider a gene for which we know the exact number of monoallelically-expressing clones, and the state of each clone is represented by flipping the coin, then what are the chances of getting a gene with all one direction of monoallelic expression? For example, in the case of three clones, the chance is 0.5 for each clone (0.5^3). This result is doubled ($2*(0.5^3)$), as there are two possible ways (all monoallelic maternal or all monoallelic paternal) of getting clones in all one direction. The result, 0.25, is multiplied by the total number of genes with exactly 3 monoallelic clones (in this case, 20) to give the expected value, 2.5 (~3).

Figure 4c shows the results from a model that attempts to most closely approximate the observed number of skewed RMAE genes, either by changing the percentage of genes subject to skewed RMAE, or by changing the probability of seeing one allele rather than the other allele. For a more detailed understanding of how each value is created, consider the individual example of the cell in the upper left corner (which has a value of 63.7). For this cell, we chose 30% of genes to choose allele A over allele B 75% of the time. For the remaining 70% of genes, if skewed RMAE were observed it would be due merely to chance, not to a true biological difference between each allele's probability of being expressed. We then calculate the number of genes expected to show skewed RMAE under these conditions and compare this value to the actual observed number of skewed RMAE genes.

Further Notes

At this point, because we lack reciprocal F1 data from the 129S1/SvImJ x Cast/EiJ cross and because the number of clones examined from Balb/cByJ x C57BL/6J reciprocal crosses was low, we are unable to distinguish between RMAE signatures that track according to parent of origin versus those that track according to strain. Future studies with a larger data set should address this question.

A

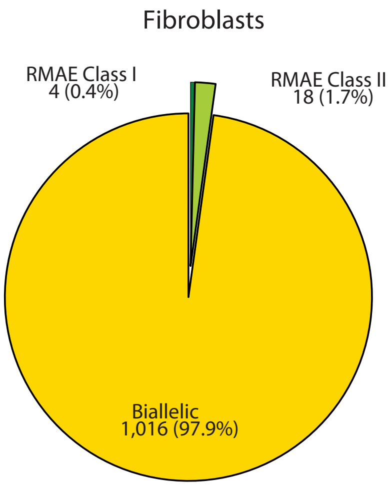

B

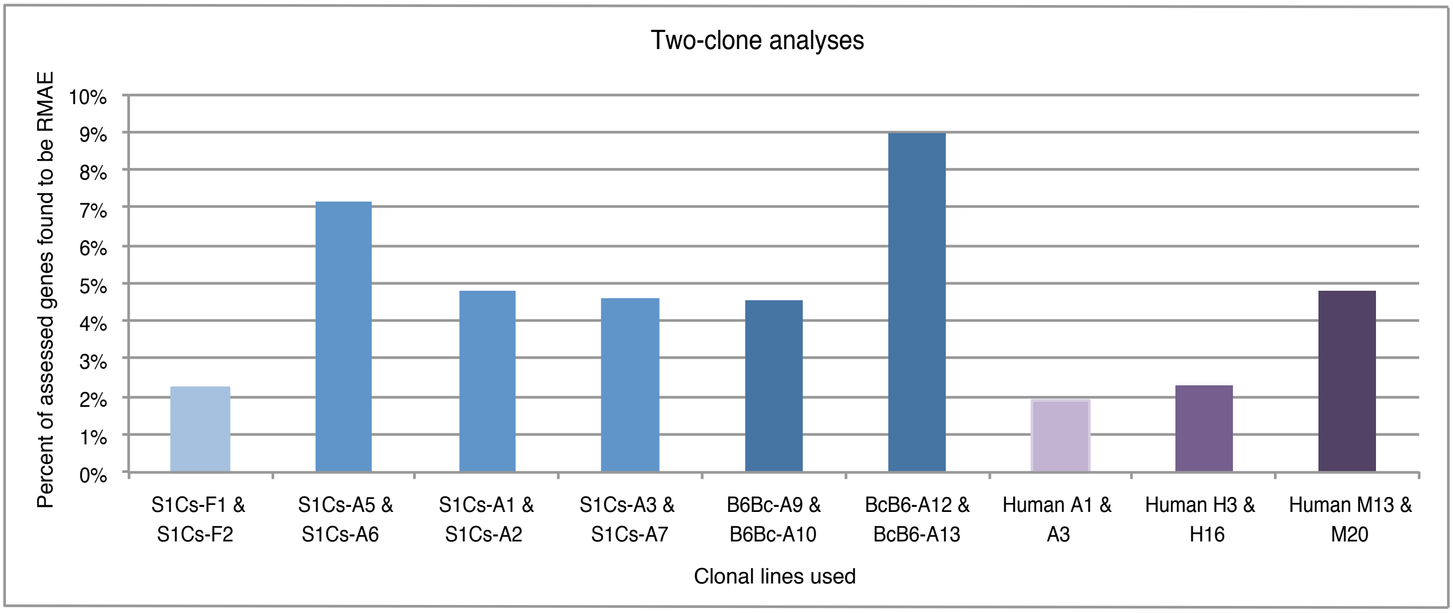

Figure S1.
(a) Assessed Fibroblasts. Yellow shows biallelic expression (BAE) genes, light green shows random monoallelic expression (RMAE) class II and dark green shows RMAE class I. In short, there appears to be less RMAE in fibroblasts than lymphoblasts, even when controlling for number of clones used in the analysis. However, this analysis is not conclusive, as the fibroblast data is based on only one set of two clones, and a more thorough comparative analysis is necessary. 763 genes were assessed in both fibroblasts and lymphoblasts. Of these, 3 were RMAE in both sets, which is about equal to the number that would be expected by chance (2.5 out of a possible 22).
(b) Comparing RMAE in different sample sets. The number of clones examined influences the efficacy of observing RMAE. To normalize for this variation, comparisons between different sample sets should involve the same number of clones. Shown above are amounts of RMAE observed in a variety of analyses, each of which uses one nonclonal and two clonal data sets.

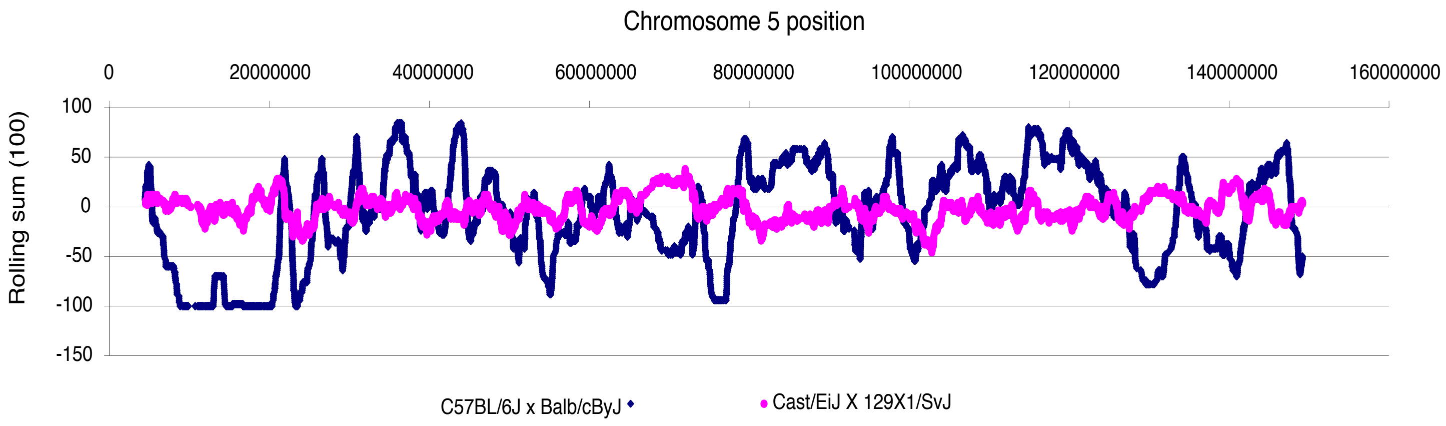

Figure S2.
Distribution of single nucleotide polymorphisms (SNPs) across chromosome 5 of two different mouse crosses. The horizontal axis shows distance in bases along chromosome 5, while the vertical axis is a rolling sum of the SNPs in a given window (non-overlapping): within a 100-SNP window, each locus receives a score of +1 if the F1 mouse genotype should be homozygous and a score of -1 if the F1 mouse genotype should be heterozygous; the sum of these values within the window is mapped. Magenta represents Cast/EiJ x 129X1/SvJ and navy blue represents C57BL/6J x Balb/cByJ.

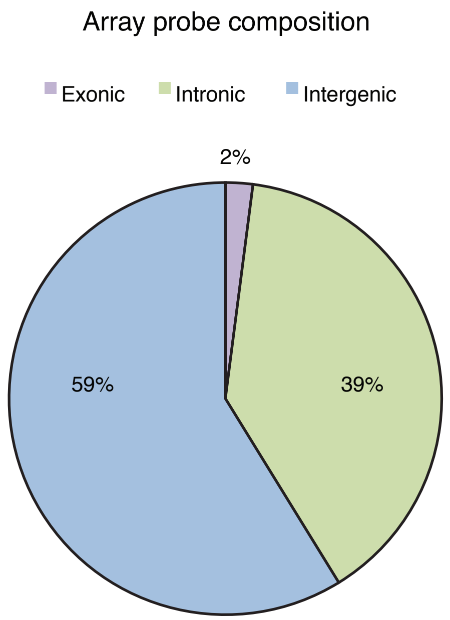

Figure S3.
The custom mouse single nucleotide polymorphism (SNP) genotyping array was meant to mimic the design of the Affymetrix Human 250K SNP Chip. As the purpose of the array was SNP genotyping, many of the array's probes are not focused in genic regions, and those that are genic comprise both intronic and exonic loci.

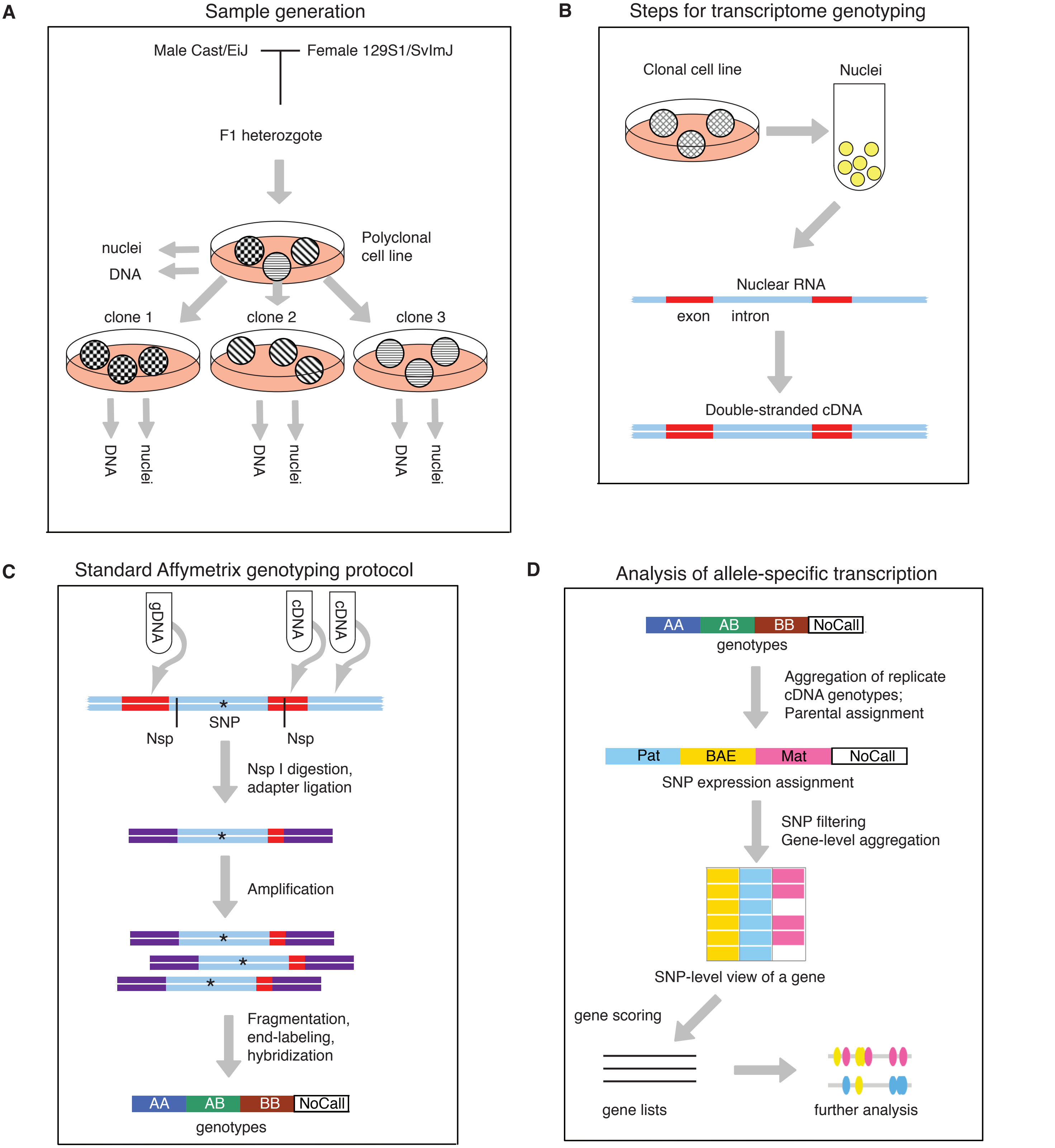

Figure S4.
Overall experimental scheme for sample and data generation. (a) Mouse lymphoblasts from various F1 were immortalized, cultured and subcloned by single cell sorting. Clones were assured to be independent by taking them from distinct mice. Genomic DNA and nuclei were purified from cells (b) RNA was extracted from nuclei and used to make random primed double stranded cDNA. (c) Genomic DNA and cDNA were processed according to standard array preparation techniques including enzymatic digestion, labeling and hybridization. (d) Calls were made using Dynamic Model Mapping Algorithm, and replicate cDNA calls were compared against gDNA to assess allele specific expression status. Filters were applied at the level of each single nucleotide polymorphism, and calls were combined to produce a gene-wide score (G-score) of random monoallelic expression status (Notes 3,4 in Additional file 1).

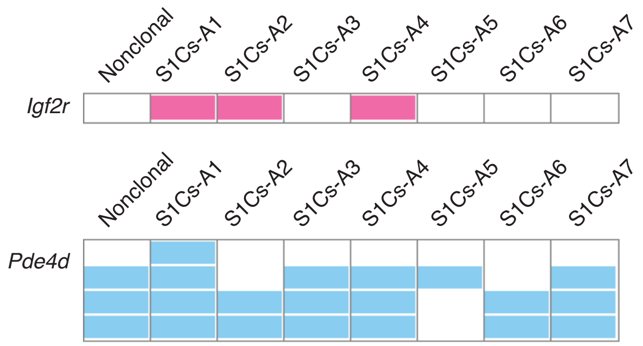

Figure S5.
Known imprinted genes (MRC Harwell) were assessed as a positive control for our assay's ability to detect monoallelic expression. Here, *Igf2r* is correctly identified as maternally expressed, and *Pde4d* is correctly identified as paternally expressed. In our search for autosomal random monoallelic expression, imprinted genes were purposefully excluded, as they constitue a form of deterministic monoallelic expression. The filters used to exclude imprinted expression patterns from our results are discussed in Additional file 1 Note 3.

Table S1. Additional information about mouse lines used in this study. An asterisk (*) indicates that a variant substrain of 129S1/SvImJ, which contained a chromosome 2 transgene, was used in this mating (see Methods).

Name	Type	Strain (MxP)	Gender	Primary Tissue Source
S1Cs-A1	Clonal Lymphoblast	129S1/SvImJ x CAST/EiJ	M	embryonic liver
S1Cs-A2	Clonal Lymphoblast	129S1/SvImJ* x CAST/EiJ	F	embryonic liver
S1Cs-A3	Clonal Lymphoblast	129S1/SvImJ x CAST/EiJ	M	embryonic liver
S1Cs-A4	Clonal Lymphoblast	129S1/SvImJ* x CAST/EiJ	F	embryonic liver
S1Cs-A5	Clonal Lymphoblast	129S1/SvImJ x CAST/EiJ	F	embryonic liver
S1Cs-A6	Clonal Lymphoblast	129S1/SvImJ x CAST/EiJ	M	embryonic liver
S1Cs-A7	Clonal Lymphoblast	129S1/SvImJ x CAST/EiJ	F	embryonic liver
S1Cs-A8	Uncloned Lymphoblast	129S1/SvImJ* x CAST/EiJ	F	embryonic liver
S1Cs-F1	Clonal Fibroblast	129S1/SvImJ x CAST/EiJ	F	adult ear
S1Cs-F2	Clonal Fibroblast	129S1/SvImJ* x CAST/EiJ	F	adult ear
S1Cs-F3	Uncloned Fibroblast	129S1/SvImJ x CAST/EiJ	F	adult ear
B6Bc-A9	Clonal Lymphoblast	C57BL/6J x Balb/cByJ	M	embryonic liver
B6Bc-A10	Clonal Lymphoblast	C57BL/6J x Balb/cByJ	F	embryonic liver
B6Bc-A11	Uncloned Lymphoblast	C57BL/6J x Balb/cByJ	M	embryonic liver
BcB6-A12	Clonal Lymphoblast	Balb/cByJ x C57BL/6J	F	embryonic liver
BcB6-A13	Clonal Lymphoblast	Balb/cByJ x C57BL/6J	F	embryonic liver
BcB6-A14	Uncloned Lymphoblast	Balb/cByJ x C57BL/6J	F	embryonic liver

Table S3. Genes assessed for X-chromosome inactivation in cell lines from female mice. Paternal refers to monoallelic paternal expression and Maternal refers to monoallelic maternal expression.

Gene	Ensemble Gene ID	S1Cs-A4	S1Cs-A7	S1Cs-F1	S1Cs-F2
Acsl4	ENSMUSG00000031278	NoCall	Paternal	Paternal	NoCall
Aff2	ENSMUSG00000031189	NoCall	Paternal	Paternal	NoCall
Arhgap6	ENSMUSG00000031355	NoCall	NoCall	NoCall	Maternal
Ar	ENSMUSG00000046532	NoCall	NoCall	Paternal	NoCall
Atp7a	ENSMUSG00000033792	Paternal	NoCall	Paternal	Maternal
Chrdl1	ENSMUSG00000031283	NoCall	Paternal	NoCall	NoCall
Col4a5	ENSMUSG00000031274	NoCall	Paternal	Paternal	Maternal
Ddx26b	ENSMUSG00000035967	NoCall	Paternal	NoCall	Maternal
Dmd	ENSMUSG00000045103	NoCall	NoCall	Paternal	NoCall
Fam120c	ENSMUSG00000025262	Paternal	Paternal	NoCall	Maternal
Fgd1	ENSMUSG00000025265	Paternal	Paternal	NoCall	Maternal
Frmpd4	ENSMUSG00000049176	Paternal	NoCall	NoCall	NoCall
Gm5127	ENSMUSG00000073010	NoCall	Paternal	NoCall	NoCall
Huwe1	ENSMUSG00000025261	Paternal	Paternal	NoCall	Maternal
Kif4	ENSMUSG00000034311	Paternal	Paternal	NoCall	Maternal
Mtcp1	ENSMUSG00000031200	Paternal	Paternal	Paternal	Maternal
Pls3	ENSMUSG00000016382	NoCall	NoCall	Paternal	Maternal
Pola1	ENSMUSG00000006678	Paternal	Paternal	Paternal	Maternal
Ptchd1	ENSMUSG00000041552	NoCall	Paternal	NoCall	NoCall
Sfrs17b	ENSMUSG00000059708	NoCall	Paternal	Paternal	Maternal
Slc16a2	ENSMUSG00000033965	NoCall	NoCall	NoCall	Maternal
Sms	ENSMUSG00000071708	Paternal	Paternal	Paternal	NoCall
Tsc22d3	ENSMUSG00000031431	Paternal	NoCall	NoCall	NoCall

Table S4. Validation Results. Validation targets were chosen by random selection of genes from either class I or class II based on G-scores generated in the 129S1/SvImJ x Cast/EiJ data set (and thus not necessarily equal to the Gmax reported in Additional file 3 Table S5). The method of validation was either Sanger sequencing or Sequenom sequencing of the same SNP locus as was originally reported on by the array. cDNA from the same F1 clones used for the array experiments was sequenced in each validation experiment, along with control gDNA from parental strains and gDNA from the clones so as to provide a baseline expectation of the dynamic range and sequencing bias found at each locus. In order to "pass" validation, monoallelic expression must have been observed in cDNA from one or more of the originally reported clones while heterozygosity of the gDNA at the same locus must also have been confirmed in the same validation assay. "Random" indicates that the gene was randomly selected from among all reported random monoallelic expression (RMAE) genes. Data on additional validated genes (chosen through non-random methods) are also presented in this table and the rationale for their selection is noted. "Skew" indicates that the gene was specifically chosen from among the pool of all genes demonstrating skewed RMAE. "Other" indicates that the gene was not chosen randomly. Lack of validation may be due either to an error in the array data or in theory it may also be due to instability of the RMAE between the two independent biological replicates, which come from separate passages of the cell culture. Successful validation of many RMAE genes indicates the stability of RMAE over time. Single nucleotide polymorphism (SNP) IDs correspond to the NCBI Build 37 of the mouse genome. RMAE class III genes that have been validated are also displayed in this table.

Gene	Class	Conclusion	Method	G-Score	Category	SNP ID
Apba1	RMAE I	pass	Sanger	1.19	Random	mm37-19-24020948
Atxn1	RMAE I	pass	Sanger	1.88	Random	mm37-13-46007070
Cobll1	RMAE I	pass	Sanger	4.5	Random	mm37-2-64968874
Galnt10	RMAE I	pass	Sanger	2.04	Random	mm37-11-57514637
Kif16b	RMAE I	pass	Sanger	3.6	Random	mm37-2-142591458
Prkcb	RMAE I	pass	Sanger	2.84	Random	mm37-7-129439136
Rasgrp1	RMAE I	pass	Sanger	3	Random	mm37-2-117139145
Slc22a15	RMAE I	pass	Sanger	2.67	Random	mm37-3-101684871
Slc39a11	RMAE I	pass	Sanger	4.5	Random	mm37-11-113158756
Ttc3	RMAE I	pass	Sequenom	1.12	Random	mm37-16-94636097
Ubash3b	RMAE I	pass	Sanger	2.25	Random	mm37-9-40884459
Zdhhc2	RMAE I	pass	Sanger	2	Random	mm37-8-41551937
Zfp704	RMAE I	pass	Sanger	1.5	Random	mm37-3-9429115
Zmynd8	RMAE I	pass	Sanger	3	Random	mm37-2-165671061
Csgalnact1	RMAE II	pass	Sanger	1	Random	mm37-8-71222751
Dock9	RMAE II	pass	Sanger	1	Random	mm37-14-122133056
Eps8	RMAE II	pass	Sanger	1	Random	mm37-6-137479159
Igf2bp3	RMAE II	pass	Sanger	1	Random	mm37-6-49100847
Klhdc1	RMAE II	pass	Sanger	1	Random	mm37-12-70363679
Klhl14	RMAE II	pass	Sanger	1	Random	mm37-18-21711078
Mast1	RMAE II	pass	Sanger	1	Random	mm37-8-87443554
Nov	RMAE II	pass	Sanger	1	Random	mm37-15-54585111
Papss1	RMAE II	pass	Sanger	1	Random	mm37-3-131300937
Poln	RMAE II	pass	Sanger	1	Random	mm37-5-34458595
Slamf6	RMAE II	pass	Sanger	1	Random	mm37-1-173868666
Cdk5rap1	RMAE II	fail	Sanger	1	Random	mm37-2-154168760
Prkch	RMAE II	fail	Sanger	1	Random	mm37-12-74720044
Nsun2	RMAE II	fail	Sanger	1	Random	mm37-13-69771140
Tfdp1	RMAE II	fail	Sanger	1	Random	mm37-8-13370321
Zfand3	RMAE II	fail	Sanger	1	Random	mm37-17-30187783
Dclk2	RMAE II	pass	Sanger	1	Skew	mm37-3-86593299
Lekr1	RMAE II	pass	Sanger	1	Skew	mm37-3-65486119
Pde10a	RMAE II	pass	Sanger	1	Skew	mm37-17-9040371
Pmepa1	RMAE II	pass	Sanger	1	Skew	mm37-2-173070978
Prss39	RMAE II	pass	Sanger	1	Skew	mm37-1-34559437
Inpp4a	RMAE I	pass	Sanger	2.22	Other	mm37-1-37387659
Bmpr1a	RMAE II	pass	Sequenom	1	Other	mm37-14-35296092
Cdk14	RMAE II	pass	Sequenom	1	Other	mm33-5-5191170, mm37-5-5364241
Myb	RMAE II	pass	Sequenom	1	Other	mm37-10-20856477
Trim66	RMAE II	fail	Sequenom	1	Other	mm37-7-116622797
Grip1	RMAE III	pass	Sequenom	0.89	Other	mm37-10-118976741
Ext1	RMAE III	pass	Sanger	0.8	Other	mm37-15-53133254
Dst	RMAE III	pass	Sequenom	0.67	Other	mm33-1-34446065, mm33-1-34384304
Bbx	RMAE III	fail	Sequenom	0.45	Other	mm37-16-50362154
